# Navicular stress fracture injury: observational use of plantar pressure measures in elite AFL footballers

**DOI:** 10.1186/1757-1146-6-S1-P11

**Published:** 2013-05-31

**Authors:** Ian North, Laurence Foley

**Affiliations:** 1 Willetton Podiatry Clinic, Willetton, Western Australia, 6155, Australia; 2 Fremantle Football Club, Fremantle, Western Australia, 6931, Australia; 3 Podiatric Medicine Unit, School of Surgery, University of Western Australia, Nedlands, Western Australia, 6009, Australia

## Background

Navicular stress fracture (NSF) is a relatively uncommon injury according to Australian Rules Football (AFL) injury surveillance data. Like all stress fractures, a disproportionate amount of compression / strain (dose) versus remodeling (response) accounts for bone breakdown. The exact mechanism of injury or anthropometric factors involved in navicular stress fracture remains largely unknown however; several authors speculate a short first metatarsal and metatarsus adductus as contributing factors. It is thought that a long second metatarsal transmits forces unevenly through the medial cuneiform creating shearing stress at the avascular central zone of the navicular. To date no prospective data is available to support these theories. Navicular stress fracture is deemed high risk due to the potential of delayed or non union and often excessive time away from activity.

## Methods

Retrospective plantar pressure mapping of two elite AFL footballers who’d sustained NSF injury were recorded. Testing involved unshod across a floor mounted pressure sensing mat (EMED SF™, Novel Munich Germany) using a two-step method following familiarisation. Five (5) trials were performed for each foot with the averages of the middle three (3) trials used for further analyses. Subsequent testing using an in-shoe pressure mapping system, (EMED ™ Pedar,Novel Munich Germany) was performed with custom moulded foot orthoses in situ. Data from twenty (20) steps were recorded, allowing for acceleration and deceleration with the middle ten (10) steps used for further analyses. Computer software generated data for Peak Pressure across 10 masked areas (kPa), Instant to Peak Pressure (ms), Contact Area (cm^2^), Force and Pressure Time Integrals were used for further anaylsis.

## Results

Analysis of the derived variables demonstrated distinct asymmetries in plantar foot loading in both subjects. Between-subject similarities exist in terms of elevated Peak Pressure (kPa) beneath the 2^nd^ metatarsophalangeal joint on the injured limb. Subject 1, demonstrates a deviation between the 1^st^ and 2^nd^ digit as a consequence of metatarsus primus adductus. Plantar pressure mapping depicted relative length differentials between the hallux and 2^nd^ digits on both subjects injured limbs. Subject 2 demonstrates asymmetry in pressure time curves between injured and non injured feet with a pronounced initial impact peak on the injured side. The implication of this in terms of injury risk is unclear.

## Conclusions

Presented is the use of plantar pressure mapping in NSF injury in two Elite AFL footballers. The results suggest increased FF pressure and anatomical variations such as a short 1st metatarsal and metatarsus adductus could contribute to NSF injury. Technology such as plantar pressure mapping may be used prospectively to assess or screen at risk groups for NSF injury.

